# Supplementary material for: Detection of Early Endothelial Dysfunction by Optoacoustic Tomography
Source: Int J Mol Sci. 2023 May 11;24(10):8627. doi: 10.3390/ijms24108627 (PMC10218068; doi:10.3390/ijms24108627)
Supplement: Supplementary file 1 [file ijms-24-08627-s001.zip › ijms-2391786-supplementary.pdf]

Supplemental Information

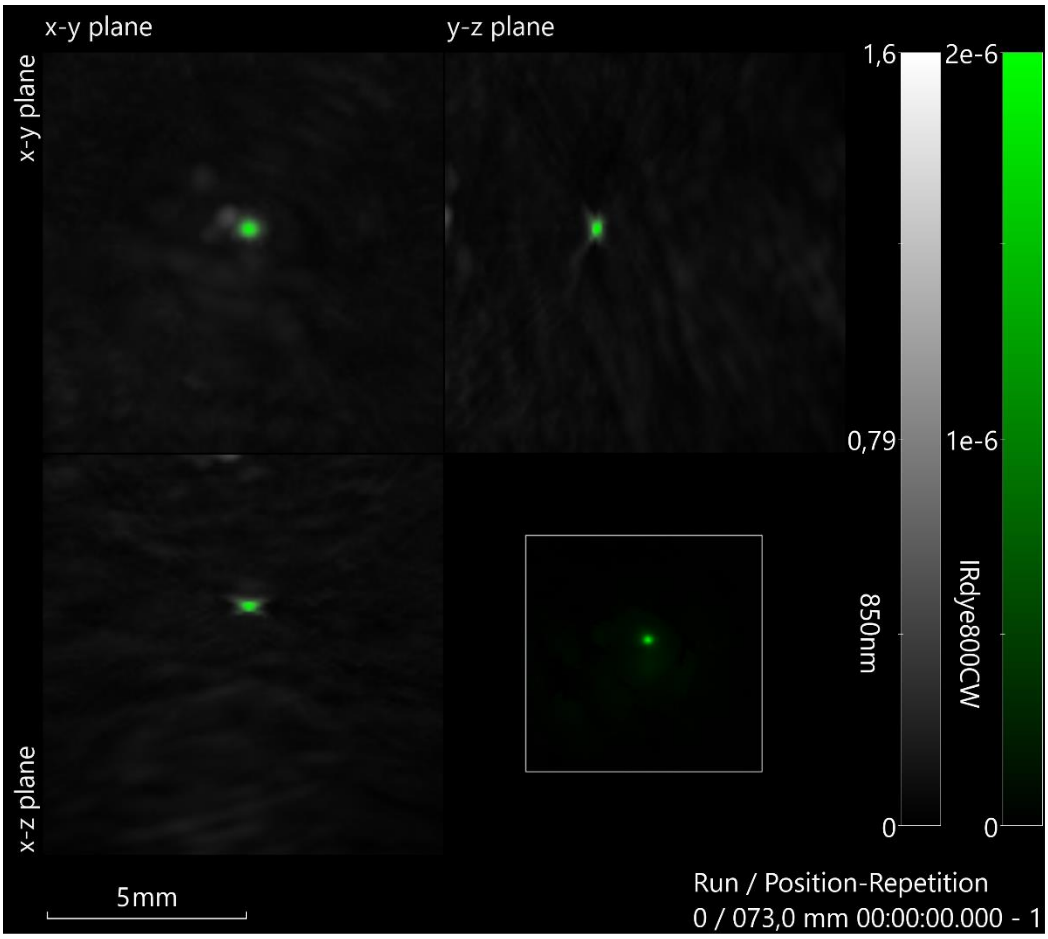

Figure S1. Optoacoustic properties of the cuff material as measured in an agarose gel phantom. The applied settings were similar to the *in vivo* experiments.
